# Depression in primary care and the role of evidence-based guidelines: cross-sectional data from primary care physicians in Germany

**DOI:** 10.1186/s12913-022-08631-w

**Published:** 2022-10-24

**Authors:** Sonia Lech, Wolfram Herrmann, Sebastian Trautmann, Ulrich Schwantes, Paul Gellert, Joachim Behr, Pichit Buspavanich

**Affiliations:** 1 Institute for Medical Sociology and Rehabilitation Science, Charité – Universitätsmedizin Berlin, corporate member of Freie Universität Berlin, Humboldt-Universität zu Berlin, Berlin, Germany; 2 Department of Psychiatry, Psychotherapy and Psychosomatics, Brandenburg Medical School Theodor Fontane, Neuruppin, Germany; 3 Department of Psychiatry and Psychotherapy, Charité – Universitätsmedizin Berlin, corporate member of Freie Universität Berlin, Humboldt-Universität zu Berlin, Berlin, Germany; 4 Institute of General Practice, Charité – Universitätsmedizin Berlin, Freie Universität Berlin and Humboldt-Universität zu Berlin, Berlin, Germany; 5 ICPP Institute of Clinical Psychology and Psychotherapy, Medical School Hamburg, Hamburg, Germany; 6 Department of Psychology, Faculty of Human Sciences, Medical School Hamburg, Hamburg, Germany; 7 Brandenburg Medical School Theodor Fontane, Neuruppin, Germany; 8 Faculty of Health Sciences Brandenburg, Joint Faculty of the University of Potsdam, Brandenburg University of Technology Cottbus-Senftenberg and Brandenburg Medical School, Potsdam, Germany; 9 Research Department of Experimental and Molecular Psychiatry, Department of Psychiatry and Psychotherapy, Charité – Universitätsmedizin Berlin, corporate member of Freie Universität Berlin, Humboldt-Universität zu Berlin, Berlin, Germany; 10 Department of Psychiatry and Psychotherapy, Gender Research in Medicine & Institute of Sexology and Sexual Medicine, Charité – Universitätsmedizin Berlin, Universität Berlin and Humboldt-Universität zu Berlin, Berlin, Germany

**Keywords:** Depression, Primary care, Mental health specialist, Collaboration, Evidence-based guidelines

## Abstract

**Background:**

Depression is the most common mental health burden worldwide. Primary care physicians (PCPs) play a key role in the care provision for people with depression. The first objective of the present study was to examine the health care situation of depression in primary care, focusing on the cooperation between PCPs and mental health specialists. Secondly, we aimed at examining the role of the German S3 Guideline for Unipolar Depression in the primary care provision.

**Methods:**

Data of N = 75 PCPs were analysed from a cross-sectional online survey. Analysis of descriptive information on the current status of primary health care and depression was conducted. Further, to examine factors that are related to the usage of guidelines, multiple regression was performed.

**Results:**

Only 22.1% of PCPs described the quality of cooperation with ambulatory mental health specialist as good. The most frequent problems in the cooperation were of structural nature (49.3%, long waiting list, few therapy units, as well as barriers in the communication and the information exchange). With regard to the role of the guideline, 65% of PCPs reported never or seldom using the guideline and 31.7% of PCPs perceived the guideline as not useful at all. In addition, perceived usefulness of the S3 guideline was positively associated with the usage of the guideline. Results of the logistic regression revealed a significant association between the usage of the German S3 Guideline for Unipolar Depression and rating of perceived usefulness of the guideline (OR: 4.771; 95% CI: 2.15–10.59; p < 0.001).

**Conclusion:**

This study highlights the central role of PCPs and demonstrates major barriers in the outpatient health care provision of depression. Present findings suggest a strong need for collaborative health care models to resolve obstacles resulting from fragmented mental health care systems. Finally, reported perceived barriers in the implementation of the German S3 Guideline for Unipolar Depression indicate the urge to involve PCPs in the development of evidence-based guidelines, in order to ensure a successful implementation and usage of guidelines in clinical practice.

## Background

Depression is one of the most common mental health conditions worldwide. According to the World Health Organization, in 2015 around 322 million people or 4.4% of the total population were affected by depression, with women being affected more often than men (5.1% vs. 3.6%) [1]. In Germany, the 12-month prevalence of unipolar depression was estimated at around 7.7% and the prevalence of major depression at 6.0% [2]. The individual and societal burden of depression is vast [1]. A recent systematic literature review on the costs of depression in Germany reported high disease-specific costs as well as high total health care expenditure [3]. In 2015, about 35% of women and 31% of men in Germany with depressive symptoms made use of psychotherapeutic or psychiatric services [4].

In Germany, outpatient mental health care is mainly provided by mental health specialists (which include psychotherapists, psychiatrists, specialists in psychosomatic medicine, and licensed clinical psychologists), as well as primary care physicians (PCP). PCPs are often the first point of contact for people with depression and play a central role in the outpatient care of depression [5, 6]. About two thirds of those affected are in treatment for depression at their PCPs [6, 7]. In Germany, a total of 59% of depression diagnoses in 2014 were made by a PCP [8] and in 2010, 64.1% of outpatient incidental depression patients received treatment and care solely by PCPs [7]. However, previous studies have reported, that treatment of depression in primary care is not optimal [6, 8]. For example, studies have shown that depressed patients in primary care are more likely to report physical symptoms (such as sleep disturbances, loss of appetite, poor concentration, and lack of energy) which are often not recognized by PCPs as depressive symptoms [9]. In addition, structural barriers in the outpatient care provision for depression are being observed. First, the German outpatient healthcare system is not a gatekeeping system: patients can consult (mental health) specialists on their own without a prior referral from their PCP [10]. Lack of cooperation between PCPs and mental health specialists was found to be one major barrier in the outpatient care of depression [11]. Further, the German outpatient mental health care is often characterized by a fragmented health care system, restricted access to mental health specialists and lack of shared-care models [11], with sometimes considerable urban-rural differences [12]. These structural factors hinder optimal outpatient care delivery for patients with depression. Due to their central role in the care of depression, there is an urgent need to study and improve outpatient mental health care for depression, with a focus on primary care.

Systematically developed, evidence-based guidelines may serve as one instrument for securing and improving individually appropriate medical care [13]. The German S3 Guideline for Unipolar Depression aims at improving the detection, diagnosis, and treatment of depression in Germany in order to optimize the care of people with unipolar depression [14]. Despite the existence of this guideline since 2009, previous empirical research suggests that around 60% of all primary care patients with depression do not receive guideline-oriented treatment with antidepressants and / or psychotherapy [6]. Psychotherapy is considered a first-line treatment alternative for mild to moderate depressive episodes and in combination with pharmaceuticals for severe depressive episodes [14–16]. However, depressed primary care patients often perceived practical barriers (for example time constraints and transportation difficulties), emotional barriers (for example discomfort talking about personal issues or stigmatization by others), or both, in the utilization of psychotherapy [17]. In Germany, the access to psychotherapy has been reported as restricted [18], and PCPs are often found to primarily prescribe antidepressants in daily routine [6]. Barriers in the utilization of psychotherapy in Germany include long waiting time [19], lack of available psychotherapists, challenging practice structures and scheduling difficulties [20] as well as personal and perceived stigmatization of psychotherapy [21]. In addition, a recent study found that provision of S3 guideline-oriented tools in primary care did not improve PCP’s attitudes towards the S3 Guideline for Unipolar Depression nor the treatment procedures [22]. However, research on the use of the S3 Guideline for Unipolar Depression and its actual impact on the quality of care for people with depression remains limited. Thus, it is of great interest to gain a better understanding of barriers in mental health care provision for depression in primary care, and to explore the role of evidence-based guidelines to evolve new solutions that aim at improving outpatient care provision for depression.

### Aim of the present study

The overall aim of the present study is to gain new insights and derive new implications for research and clinical practice of outpatient care for depression, in particular for primary care. The key objective of the present study was twofold. First, to examine the current state of outpatient collaboration between PCPs and outpatient mental health specialist, such as psychiatrist and psychotherapists in the care of depression, as well as to identify barriers that hinder a fruitful collaboration. Based on previous literature, we expected the collaboration to be poor and assumed a variety of different barriers harming a successful cooperation. The second objective was to examine the role of the German S3 Guideline for Unipolar Depression in primary care. This included PCPs knowledge about and usage of the guideline, perceived usefulness of the guideline as well as perceived barriers in the implementation of the guideline in clinical practice. Further, to explore barriers of guideline implementation and factors associated with the guideline usage, the association between PCPs usage of the German S3 Guideline for Unipolar Depression and variables on PCPs level such as age, sex, years of experience as a PCP, number of patients with depression, and ratings of usefulness of the guideline were explored.

## Methods

### Setting, study design and sample

**Figure 1:**
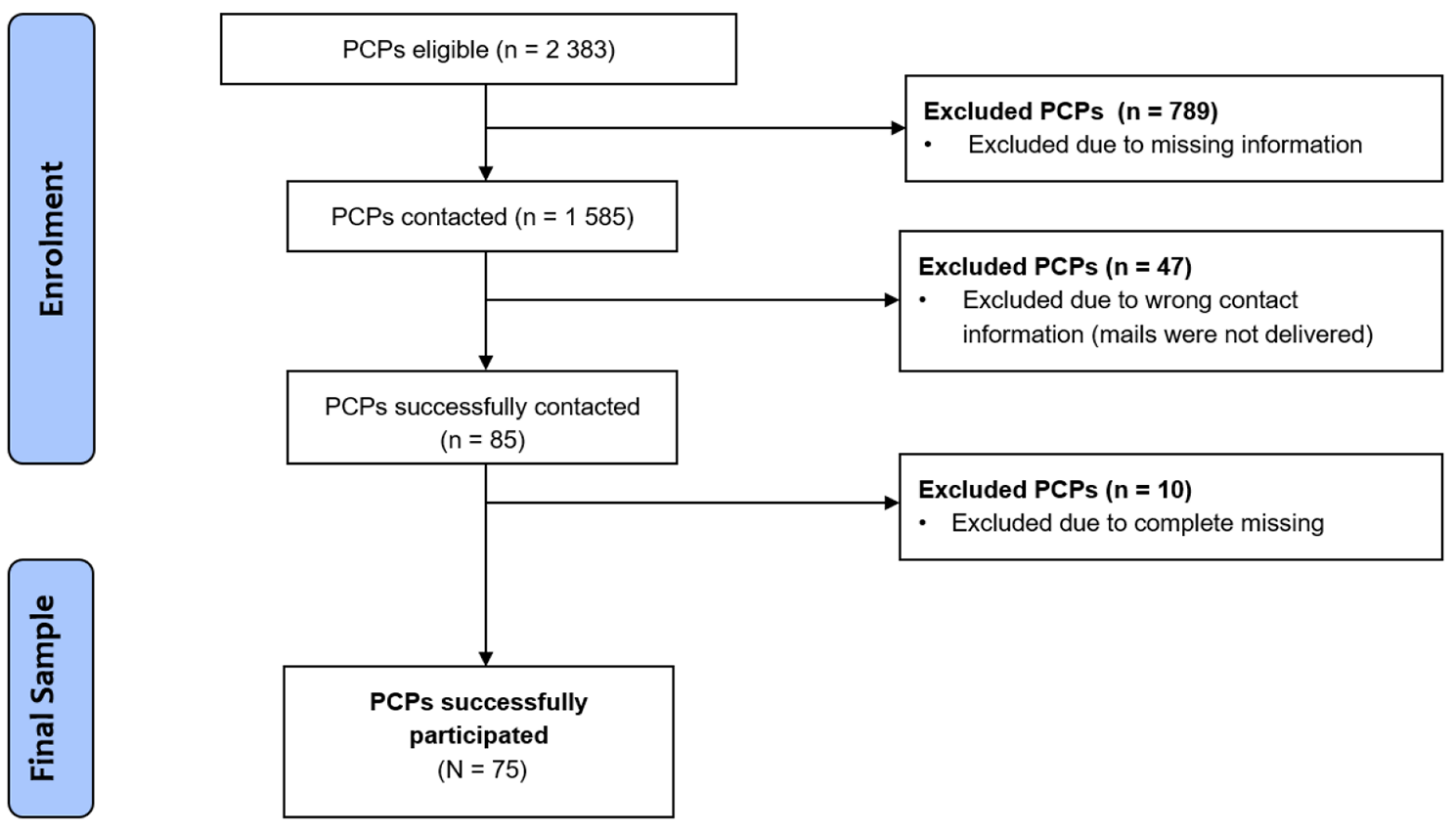
Flow chart of the recruitment.

The present paper used data obtained from the DeCare Study, an anonymous online cross-sectional survey conducted among PCPs from Berlin and lager parts of Brandenburg. The survey was administered in German language. Participation was anonymous, voluntary and without any compensation. All participants were asked to review information on the research project and provide consent prior to data collection. The survey was registered by the Ethics Review Committee of the Medical School Brandenburg (registration number: E-01-20200309). The study was performed in accordance with the Declaration of Helsinki. For the recruitment of PCPs, digital search engines of a data base provided by the Statutory Health Insurance Physicians (Kassenärztliche Vereinigung) in Berlin and Brandenburg were used. Inclusion criteria included (1) formation in general practice and (2) knowledge of German. With the help of previously determined postcodes, lists of eligible PCPs were created and contact details reported. Missing contact information was obtained using the Google search engine. The database search yielded a total of n = 2 383 PCPs. However, due to missing contact information, a total of n = 798 PCPs could not be contacted. Between July 2020 to October 2020 a link to the survey was send to a total of N = 1 585 eligible PCPs via Email, followed by one follow-up reminder after six weeks. For n = 47 PCPs the contact information found during the search was not correct and the mail was not delivered to PCPs. A total of n = 85 PCPs participated in the study. Due to missing values, n = 10 participants had to be excluded from the analysis. Eligible participants for analysis resulted in N = 75 participants. This represents a recruitment rate of 4.7%, which is comparable to previous research [23]. A flow chart can be found in Fig. 1.

### Measures

*Sociodemographic information*: characteristics of PCPs included age (years), gender (female/male/other), type of practice (single/shared), years of experience as PCP (years), additional training in psychotherapy (yes/no), number of depressive patients seen in their practice during last three months (NumPwD), age group of patients with depression (20–30 years, 31–40 years, 41–50 years, 51–64 years, 65 or more years), severity of depression (mild, moderate, severe depression) among patients seen in their practice, and number of visits of depressive patients during the last three months. The evaluation of the depression diagnosis was assessed with the question: *“How do you assess a diagnosis of depression with your patients?”* and the following answers: based on (1) clinical opinion, (2) patient’s history, (3) ICD-10 criteria and (4) standardized assessments (such as the Beck´s Depression Inventory, Patient Health Questionnaire, Hamilton Depression Scale, Geriatric Depression Scale, Depression Screening Questionnaire or Well-Being Index).

*Cooperation between PCPs and specialists*: the cooperation between PCPs and specialists (psychiatrist or psychotherapists) was assessed with the following variables: collaboration frequency (“*How many of your patients receive specialist care?*”), type of cooperation (“*With which specialists do you collaborate*?”, psychiatrist/psychotherapist/both), rating of cooperation (“*How would you rate the cooperation between you and specialists (psychiatrists, psychotherapist, other)?”, very good/good/medium/bad/very bad)*, and problems with the cooperation (“*What problems arise in the cooperation with psychiatrists?”, open-ended item).*

*Experience with the German S3 Guideline for Unipolar Depression*: the role of the German S3 Guideline for Unipolar Depression in primary care was assessed using the following items: knowledge of guideline (*“Are you familiar with the guideline?”, yes/no)*, usage of guideline *(“How often do you use the guideline?”, never/seldom/sometimes/often/always)*, usefulness of guideline *(“How useful do you rate the guideline?”, not at all/somewhat/partially/very useful)*, barriers for implementation (*“Which barriers/problems do you see in the implementation of the guideline for your practice?”, open-ended item)* and improvements in guideline development *(“How could the guideline be developed, so that you would find it more useful?”, open-ended item).* Answers provided by PCPs for each item were analyzed and coded.

### Statistical analysis

Responses provided for the open-ended items were analyzed applying quantitative content analysis [24]. First, based on responses, a list of categories for each item was derived by two researchers. Second, responses were systematically categorized and transformed into quantitative data applying a coding system (one code per category) by two independent coders using Microsoft Excel 2019. Answers in multiple categories per person were possible. Finally, to ensure reliability of the coding, a random sample of item responses (10% for each item) was drawn from the data set and codes were independently proofed by a researcher. Descriptive analyses (means, standard deviations and ranges for continuous variables, and frequencies for nominal and ordinal variables) of the variables of interest were analyzed. In order to examine the association between guideline usage (dependent variable as binary variable) and predictors on PCP level (age, sex, experience as a PCP, number of patients with depression (NumPwD) and rating on usefulness of the guideline) a multiple logistic regression was performed. All analyses were performed using IBM SPSS Statistics for Windows, V.27.0. All tests of significance were based on p < 0.05 level and a confidence interval of 95% was applied.

## Results

### Sample characteristics

Main characteristics of PCPs and their patients with depression can be found in Table 1. Overall, 62.3% of PCPs were female and on average 53.7 years old (*SD* = 9.0, range: 33–80 years), with a mean of about 16.5 years of experience as a PCP (*SD* = 10.5, range: 1–44 years) and only 5.4% of PCPs reported a special training in psychotherapy. The majority of PCPs (n = 52, 70.3%) were working in a single-handed practice. On average, PCPs had treated N = 115.2 patients with depression (*SD* = 136.3, range: 5–800 patients with depression) in the past three months. On average one patient visited the PCPs practice almost five times within the last three months (*SD* = 3.4, range: 1–200 times) and approximately one quarter (26.5%) where over the age of 65 years, who can be considered geriatric patients with potentially more somatic comorbidities. According to PCPs, less than the half of their patients (42.7%) receive specialist mental health care. In terms of diagnostics, 59.9% of PCPs reported conducting the diagnostics of depression based on their clinical opinion, 66.2% based on the patient’s history, 40.5% based on ICD-10 criteria and 14.9% based on standardizes assessments.

**Table 1:** Main characteristics of PCPs and their depressed patients

	n*	%	*M*	*SD*	range
*PCPs*					
Age	74		53.7	9.0	33–80
gender (female)	45	60.8			
*Years of experience as PCP (years)*	74		16.5	10.5	1–44
Training in psychotherapy	4	5.4			
Single handed practice	52	70.3			
Patients with depression					
NumPwD	68		115.2	136.3	5–800
Age of PwD	68				
20–30 years	1	1.5			
31–40 years	5	7.4			
41–50 years	21	30.9			
51–64 years	23	33.8			
65 or more years	18	26.5			
Most frequent depression type	68				
mild depression	24	35.3			
moderate depression	43	63.2			
severe depression	1	1.5			
PwD in specialist treatment	65	42.7			
Frequency of PCPs visits during three months	57		4.6	3.4	1–20

Note: N = 75, * = Number of PCPs varies due to missing data; N PCP = Primary care physicians, M = Mean, SD = Standard deviation, NumPwD = total number of patients with depression seen during the last three months. PwD = Patients with depression

### Cooperation between PCPs and mental health specialist

Table 2 describes the cooperation between PCPs and mental health specialists. While most of PCPs work together with at least one psychiatrist, only less than 5% of PCPs work together with a psychotherapist. Further, 41.2% of PCPs describe the quality of cooperation with ambulatory specialists as medium. While 29.4% describe the quality of cooperation as good, 22.1% describe it as bad.

**Table 2:** Cooperation between PCPs and ambulatory specialists

	*N*	%
*Type of cooperation*	64	
psychiatrist	49	76.6
psychotherapist	3	4.7
both	12	18.8
*Rating of cooperation*	68	
very good	1	1.5
good	20	29.4
medium	28	41.2
bad	15	22.1
very bad	3	4.4

**Figure 2:**
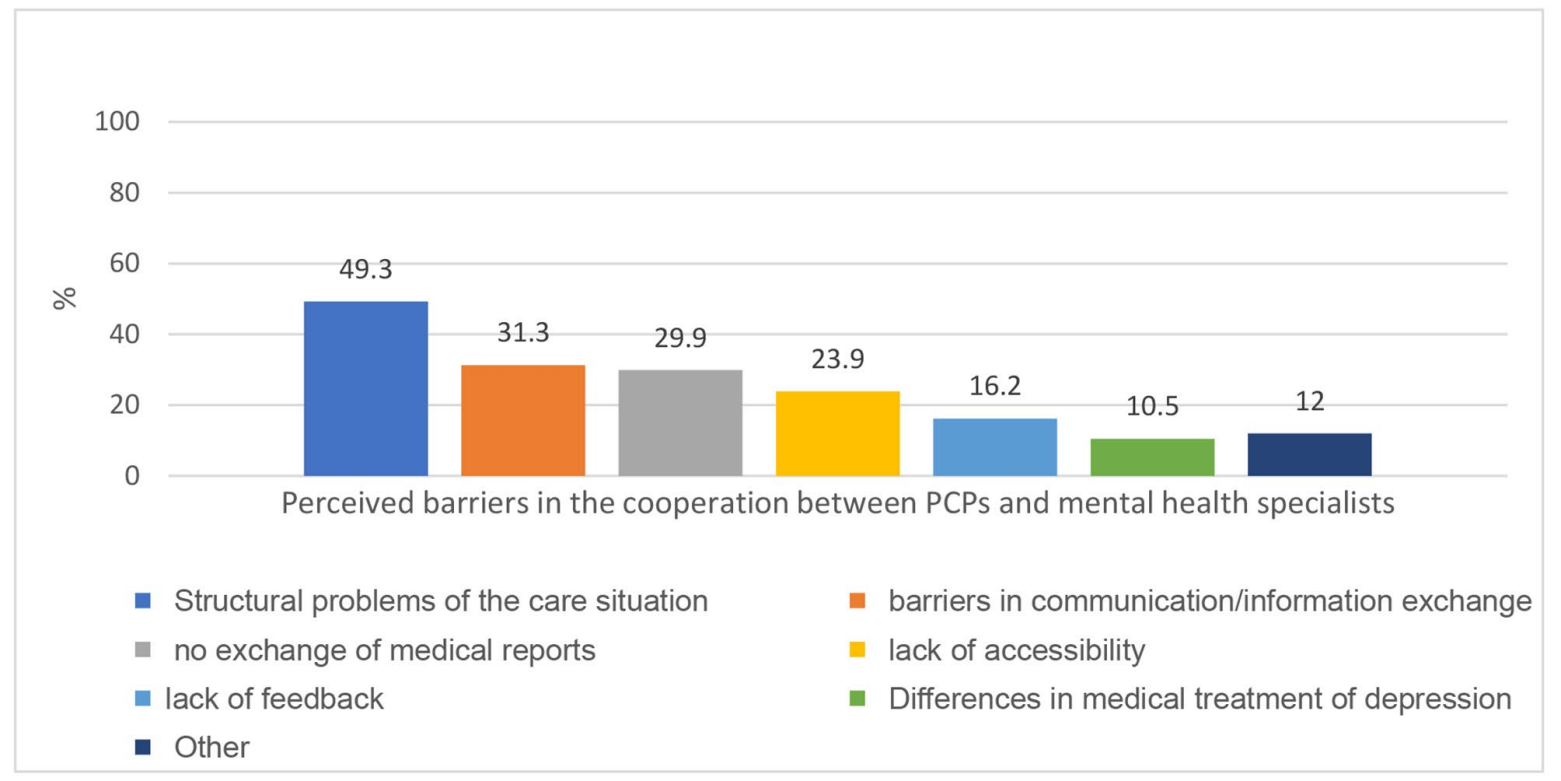
Categories drawn from data on perceived barriers in the cooperation between PCPs and mental health specialists (%), multiple categories per PCP possible, N = 67

Figure 2 summarizes the answers and categories of open-end questions regarding perceived difficulties in the cooperation with mental health specialists. The most frequent problems in the cooperation were structural problems (49.3%) which included long waiting lists, few therapy units, as well as barriers in the communication and the information exchange between PCPs and specialists (31.3%), and a lack of medical report exchange between PCPs and specialists (29.9%)

*The role of the German S3 Guideline for Unipolar Depression*.

**Figure 3:**
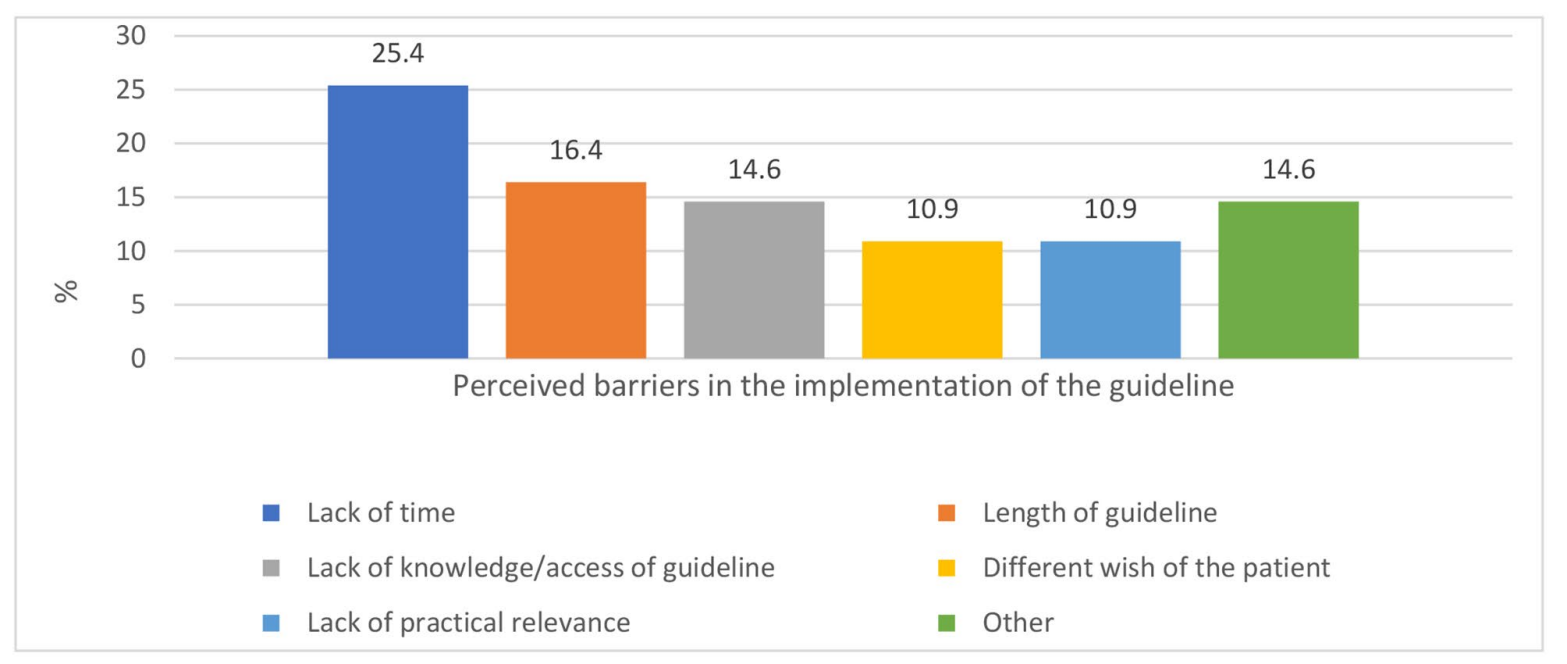
Categories drawn from data on perceived barriers in the implementation of the guideline (%), multiple categories per PCP were possible, N = 60

Table 3 describes the role of the guideline in the care of depression in primary care. Half of PCPs (50%) reported knowing the German S3 Guideline for Unipolar Depression. However, the great majority of PCPs reported never or seldom using the guideline (65%). One third (31.7%) perceived the guideline as not useful at all. Figure 3 shows categories drawn from responses with regard to PCP’s perceived barriers in the implementation of the guideline. Perceived barriers included lack of time (25.4%), length of the guideline (16.4%) as well as lack of knowledge and/or access to the guideline (14.6%). Barriers of implementation are presented as categorized answers to open-end questions.

**Table 3:** The role of the German S3 Guideline for unipolar depression in primary care

	N	%
*Knowledge of the guideline (yes)*	30	50.0
*Frequency of the guideline usage*	60	
never	25	41.7
seldom	14	23.3
sometimes	9	15.0
often	10	16.7
always	2	3.3
*Usefulness of the guideline*	60	
not at all	19	31.7
somewhat	14	23.3
partially	14	23.3
very	13	21.7

### Association between usage of guideline and PCPs individual characteristics

Results on the link between individual PCPs characteristics and the usage of the German S3 Guideline for Unipolar Depression can be obtained from Table 4. Results of the logistic regression revealed a significant association between the usage of the German S3 Guideline for Unipolar Depression and the rating of perceived usefulness of the guideline. All other covariates were not significantly related to guideline usage.

**Table 4:** Multivariate logistic regression model for the association between the usage of the guideline and PCPs individual characteristics

	Regression coefficient	Standard Error	p - value	95% Confidence Interval Lower Upper	
Age (in years)	0.977	0.079	0.769	0.837	1.141
Gender (female)	0.547	0.780	0.439	0.118	2.525
Years of experience as PCP	0.996	0.074	0.953	0.861	1.151
NumPwD	1.003	0.002	0.233	0.998	1.008
Usefulness of guideline	4.771	0.407	< 0.001	2.150	10.589
**Statistics for logistic regression**					
Nagelkerke’s R^2^	0.549				
Standard Error	0.263				

Notes: PCP = Primary care physicians; p = p-value; NumPwD = total number of patients with depression seen during the last three months

## Discussion

The first objective of the present study was to examine the role of evidence-based guidelines for depression in primary care. In particular, we focused on the cooperation between PCPs and mental health specialists such as psychiatrists and psychotherapists. Previous research has already acknowledged the central role of PCPs in the diagnosis, treatment, and care of depression. Generally, results of the present study underline the key role of PCPs in health care provision for depression as well as a lack of cooperation between PCPs and mental health specialists, in particular the importance of communication and exchange between PCPs and mental health specialists. Secondly, we aimed at examining the role of the German S3 Guideline for Unipolar Depression in the primary care provision. PCP’s personal attitude (perceived usefulness of the guideline) was positively associated with the usage of the German S3 Guideline for Unipolar Depression in daily practice.

### PCPs and collaboration with mental health specialists.

Present data indicate that PCPs see on average about 115 patients with depression per quarter, which represents about 38 patients with depression per month or 1.3 patients with depression per day. The central role in the diagnosis, treatment and care provision of PCPs has already been acknowledged numerous times [25–27]. In Germany, outpatient mental health care of depression is mainly provided by PCPs and mental health specialists. However, present results indicate major barriers in and lack of cooperation between PCPs and mental health specialists, in particular with psychotherapists. Almost half of participating PCPs described the quality of cooperation with ambulatory specialists as medium, indicating the need for further improvement of collaborations. This finding is in line with previous research [11, 28, 29]. For example, a recent cross-sectional study on the collaboration between PCPs and psychologists reported that the vast majority of participating psychologists (64%, n = 278) found the cooperation unsatisfactory [30]. Main problem areas included poor communication and lack of time. Lack of communication and information exchange between PCPs and mental health specialists has been acknowledged numerous times in the past [31, 32], particularly with psychotherapists [33]. For example, a German study examining reply letters of specialists to PCPs found low numbers of reply letters and a need for improvement in the communication between PCPs and specialists [34]. Further, the number of specialist’s reply letters varied across specialists and was found to be lowest for, among others, psychiatrists [34]. In a recent study among PCPs, psychiatrists, and psychotherapist, participants reported a lack of structures and routines for cooperation among mental health care providers and a great need for improvement of collaboration [35]. This is in line with our findings. Furthermore, we reported that the most commonly perceived barriers to the usage of guidelines were lack of time and length of the guidelines. This finding is not surprising, given that previous studies reported similar barriers including lack of time, communication problems, and lack of established structures for collaboration. However, overall participants reported moderate satisfaction with their existing cooperation [35]. Further, the positive impact of interprofessional and collaborative care is not only reported by health professionals, but patients also report (health) benefits [32]. To sum up, interprofessional collaboration for effective primary health care has long been suggested as essential [36]. However, management of mental health disorders such as depression in primary care is complex [37] and the translation of collaborative care models into daily practice continues to be challenging [38, 39]. Interestingly, although half of PCPs (50%) reported knowing the German S3 Guideline for Unipolar Depression, the vast majority of PCPs reported never or rarely using the guideline (65%). Furthermore, as perceived barriers against the usage of the guidelines, participants reported structural problems of the care situation and barriers in communication/information exchange. These findings point towards a need for the establishment of new structures and collaboration models to increase patient-oriented outpatient care for patients with depression. To investigate optimal conditions for a successful implementation of evidence-based structures, future research should focus on interventional studies that aim at examining the effectiveness of collaborative care models between PCPs and mental health specialists as well as facilitators and barriers in their implementation. This might also include, in a first step, educational trainings for PCPs to strengthen their skills and comfort level in the management of depression and to improve the collaboration among primary care physicians [40].

### Guideline recommendations for Unipolar Depression and primary care

Present data show that knowledge, perceived usefulness and usage of the guideline is restricted among PCPs in Germany. These findings are partially in line with previous research [6], where, similar to our results, only 41% of PCPs reported knowing the guideline recommendations for the treatment of depression. However, in contrast to our study, participating PCPs considered the guideline to be generally useful in their daily practice. Based on present findings, the central question arises, how the implementation and usage of evidence-based guidelines in primary care can be improved? Although the German Association for Primary Care was involved in the development of the German S3 Guideline for Unipolar Depression, the guideline was majorly developed by mental health specialists such as psychiatrists and psychotherapists. Drawing on present data, which show that PCPs do not know about the guideline or do not use it and do not perceive it as useful, future research should investigate effective mechanisms of guideline implementation in primary care. Further, in Germany, PCPs have on average about 7.6 min per patients [41]. This is rather low compared to other European countries such as Sweden (22.5 min), Bulgaria (20 min) or Norway (20 min) [41]. Present results suggest a lack of time as a main reason for the non-use of the German depression guideline by PCPs and past research has found that longer consultations result in better diagnosis and treatment of mental health problems such as depression [42]. Therefore, it is assumable that a lack of time negatively impacts the treatment of depression in primary care, as depression represents a health problem that requires more consultation time, especially during the first consultation [43].

As adherence to evidence-based guidelines may improve quality of care for depression [44], the present study aimed at examining factors that are related to the usage of the guideline. Results showed, that perceived usefulness of the guideline was positively associated with the usage of the guideline. These findings are partially in line with previous studies. In addition, and as reported by participating PCPs, the German S3 Guideline for Unipolar Depression is quite long and lacks practically relevant recommendations. We highly recommend designing the next guideline in a way that PCPs, who are often the first point of contact for patients with depression, perceive it as useful and relevant. To ensure the implementation of evidence-based guidelines in clinical practice, perspectives, and experiences of important stakeholders such as PCPs should be integrated in the development and dissemination of guidelines. Further, it is important to address barriers in the implementation of evidence-based guidelines in primary care [45]. Future research should further explore successful implementation strategies for newly developed guidelines, in particular for primary care. Currently, in Germany, a new national guideline for the treatment of depression is being developed. Based on present findings, we strongly recommend including PCPs perspectives in the development of the new German depression guideline. However, when examining the role of guideline-based depression care in primary care, future research should include patient-oriented health outcomes. Although the potential of evidence-based guidelines for the improvement of health care is well described, adherence to specific guidelines does not necessarily indicate best quality of care provided for an individual.

### Limitations

There are a number of limitations that must be outlined. First, due to the cross-sectional design we cannot draw conclusions about causal relationships. Second, although the response rate of the present study is comparable to previous primary care research [23, 46], the overall response rate was rather low. This leads to limitations in the generalizability of the sample. The present sample is based on a convenience sample as PCPs self-selected themselves into the study. Self-selection may propose a higher risk of biased data, as participants’ decision to participate may be linked with traits that affect the results of the study [47]. As participation was voluntary and most contacted PCPs did not participate in the survey, it may be that only PCPs who were specifically interested in or experienced with treatment and care of depression participated. The self-selection bias is a well-known problem in research, as self-selection has been shown to impact the validity of results [48]. Comparing present sociodemographic characteristics of participating PCPs with previous, representative PCP samples, demographic characteristics of the present sample, for example age, are comparable to some extent [49]. Further, only four of the participating PCPs reported having attended a separate training on psychotherapy, which might diminish the assumption that only PCPs who are particularly interested and skilled in mental health participated. However, a strong risk of a selection bias must be taken into account when interpreting the results, which represents a major limitation of the present study. Furthermore, aside from age groups, the present study has not assessed socio-demographic information of PCP’s patients with depression. Patients’ characteristics may influence the treatment and care provision of PCPs. In addition, the present study only focused on two geographical areas (Berlin and the surroundings) and only represents the perspectives of PCPs. Future research should aim for a more representative sample in terms of both region and population, and include perspectives of mental health specialists. For example, in order to gain a more comprehensive understanding of outpatient and primary care of depression, focus groups and interviews with all outpatient health care providers and patient representatives could be of great value.

## Conclusion

The present study provides novel insights into the role of PCPs in mental health care provision. Further, findings demonstrate a variety of barriers in the collaboration between PCPs and mental health care specialists. We conclude a strong need for collaborative health care models instead of fragmented mental health care systems. Finally, moderate usage and critiques of PCPs regarding the current format of the German S3 Guideline for Unipolar Depression indicate a need to improve evidence-based guidelines and include PCP’s perspectives and experiences, to ensure a successful implementation and usage of evidence-based guidelines in primary care practice. Furthermore, the specific needs of PCPs should be considered, such as the fact that patients may present with more physical symptoms (such as sleep disturbances, loss of appetite, poor concentration, and lack of energy) that require different diagnostic measures.

## Data Availability

Data are stored in a non-publicly available repository. Data are however available from the corresponding author on request.

## List of abbreviations

PCP: primary care physician.
M: mean.
NumPwD: total number of patients with depression seen during the last three months.
SD: standard deviation.
